# Iron overload in myelodysplastic syndromes (MDS) – diagnosis, management, and response criteria: a proposal of the Austrian MDS platform

**DOI:** 10.1111/j.1365-2362.2007.01915.x

**Published:** 2008-03

**Authors:** P Valent, O Krieger, R Stauder, F Wimazal, T Nösslinger, W R Sperr, H Sill, P Bettelheim, M Pfeilstöcker

**Affiliations:** * Medical University of ViennaAustria; † Elisabethinen HospitalLinz, Austria; ‡ Innsbruck Medical UniversityAustria; § Ludwig-Boltzmann Institute for Leukemia Research and Hematology and 3rd Medical Department, Hanusch HospitalVienna, Austria; ¶ Medical University of GrazAustria; ** Otto Wagner Hospital ViennaAustria

**Keywords:** Chelation, ferritin, HFE, iron overload, response criteria, transfusions

## Abstract

Transfusion-related morbidity is an emerging challenge in chronically transfused patients with low-risk myelodysplastic syndromes (MDS). In these patients, transfusion-induced iron overload may represent a leading medical problem. However, although iron-chelating drugs are available, little is known about optimal diagnostic tools, predisposing factors, and the optimal management of these patients. In the current article, we provide recommendations for the diagnosis, prevention and treatment of iron overload in MDS and propose treatment response criteria. Consensus criteria and resulting recommendations were discussed and formulated by members of the MDS platform of the Austrian Society of Haematology and Oncology in a series of meetings and conferences in 2006 and 2007. These recommendations should facilitate and assist in recognition of iron overload, selection of patients, timing of treatment, drug selection and the measurement of treatment responses.

Eur J Clin Invest 2008

## Introduction

Myelodysplastic syndromes (MDS) are a heterogeneous group of myeloid neoplasms defined by abnormal differentiation and maturation of myeloid cells, bone marrow failure, and a genetic instability with an enhanced risk of transformation to secondary leukaemia [1–5]. Most patients with MDS present with transfusion-dependent anaemia with or without additional cytopenias [1–4]. MDS are classified according to their pathogenesis (*de novo* or *secondary*), cytological features, and specific karyotypes. A most useful classification system has been the proposal of the French-American-British (FAB) cooperative study group [6,7]. More recently, the World Health Organization (WHO) has worked out an updated classification [8] that represents an extension of the FAB proposal, with several modifications, that include the removal of the subvariant ‘refractory anaemia with excess of blasts in transformation’ (RAEB-T; now in the group ‘acute myeloid leukaemia’, AML) and of chronic myelomonocytic leukaemia (CMML), recognition of the impact of multilineage dysplasia, and delineation of a cytogenetically defined variant, the 5q-syndrome [3,4,8]. However, in one particular WHO category, the prognosis and clinical picture may vary among patients depending on deregulated genes and the specific biological properties of the clone(s) [3–6]. Whereas some patients transform to leukaemia or die from complications of bone marrow failure within a short time, other patients survive for many years. The International Prognostic Scoring System (IPSS) and other scoring systems (like the Düsseldorf-score, Toyama-score, Mufti-score or Sanz-score) can assist in prognostication in MDS concerning survival and leukaemia-free survival [3–5,9]. All these score systems are based on multiple prognostic parameters.

In patients with low-risk MDS and transfusion-dependent anaemia, one important clinical feature is iron overload which develops in most cases [3,10,11]. Some of these patients develop massive iron overload over time with consecutive organopathy (hepatopathy, cardiomyopathy and others) despite chelation therapy [10–12].

A number of different strategies aimed at counteracting iron overload in MDS have been proposed. A straightforward approach is to introduce iron-chelating agents such as desferoxamine, deferiprone (L1) or deferasirox (ICL670) [11,12]. Another approach is to prevent blood transfusions and thus iron overload by early therapeutic intervention with growth factors (erythropoietin with or without additional granulocyte colony-stimulating factor, G-CSF) or specific targeted drugs. In addition, current efforts are being made to define genetic factors that predispose to, and thus predict, rapid iron overload [13,14]. However, although several study groups have started to discuss criteria and recommendations [15–17], little is known about optimal diagnostic tools, predisposing factors and the optimal management of iron overload in these patients. In addition, most of these proposals lack suitable response criteria. During the past 12 months (October 2006–October 2007), an expert panel of the MDS platform of the Austrian Society for Haematology and Oncology has worked out definitions, criteria and recommendations for chronically transfused patients with MDS (based on literature data) in a series of panel-meetings. The respective outcome and consensus are reported herein.

## Diagnosis, classification, and prognostication of MDS

When considering iron overload and the potential use of chelating agents, it is of importance to establish the diagnosis and variant of MDS, and to estimate survival and AML-free survival in these patients. Likewise, chelation therapy cannot be regarded as an important therapeutic manoeuvre in patients who are considered to transform to AML within a short time or are scheduled to receive chemotherapy.

Diagnostic criteria for MDS are available and should be applied in all patients [3,6–8]. For estimation of survival and AML-free survival, the IPSS is recommended as a gold standard of prognostication [9]. Other scoring systems and additional prognostic factors (not included in the IPSS such as LDH or age) may also be employed [18–23]. A clear advantage of the IPSS over other older scoring systems is that it also includes cytogenetic features, which appear to be of major prognostic significance [9]. With regard to iron overload, the recently proposed WPSS score may be of particular interest as this score includes the transfusion frequency (apart from cytogenetics and blast cells) as a prognostic variable and may also be used in the follow-up [23]. However, because of its general use and validation in numerous studies, the IPSS is still considered as gold standard in prognostication in MDS.

## Definition of iron overload and recommended investigations

To determine and quantify iron overload, several different investigations have been proposed [24–30]. These investigations include serum ferritin levels, transferrin saturation, liver and cardiac imaging studies, and tissue histology (preferred: bone marrow, as this is often available; other principle options: liver or gastric mucosa) [24–30]. We recommend to assess the serum ferritin level and transferrin saturation together with liver enzymes and inflammation markers such as fibrinogen or C-reactive protein = CRP as a simple (and non-invasive) initial diagnostic step, in order to determine or exclude iron overload. A constant increase of ferritin exceeding 2000 ng mL^−1^ should be considered as a relatively safe indication of iron overload provided that no severe coexisting active systemic inflammatory or active liver disease is present. Without effective therapy, ferritin levels and thus the iron load may further increase in chronically transfused MDS patients, and may cause severe clinical problems, and even organ failure of, for example, the heart and liver.

As mentioned, an important limitation is that in the case of an ongoing inflammation, the ferritin level may also increase. In these patients, the test should be repeated in a symptom-free interval. If this is not possible because of constant chronic inflammation, a liver imaging test [recommended: Magnetic Resonance Imaging (MRI) or Superconducting QUantum Interference Device (SQUID)] should be considered [27–30]. In addition, it is important to repeat ferritin levels in certain time intervals (every one to three months, depending on the severity of iron overload, dynamics in ferritin-increase, response to drugs and the overall situation of the patient) in these patients to confirm or to exclude a further increase in the body iron burden. A liver biopsy is not recommended in these patients because of the risk of bleeding. However, it may be helpful to re-assess the bone marrow biopsy material, if available, and ask for signs of tissue iron overload. The value of gastric mucosa biopsies in the evaluation of iron overload in MDS has not been validated so far.

A number of different genetic polymorphisms and mutations are known to predispose to iron overload [31–34]. A good example is distinct *HFE* gene mutations, primarily C282Y and H63D. Homozygosity is often associated with frank haemochromatosis [31,32]. In contrast, the clinical significance of heterozygosity and of mutations (polymorphisms) in genes other than *HFE* are not well established. However, it is assumed that heterozygosity for *HFE* gene mutations, or other mutations, could predispose to iron overload, which can manifest when additional predisposing factors, such as chronic transfusions, are present [33,34]. Other gene products that have been implicated in the regulation of iron metabolism and storage are ferroportin 1 (FPN1), hemojuvelin (HFE2), and hepcidin (HAMP) [32].

More recently, it has been described that *HFE* gene polymorphisms (mutations) are frequently detected in patients with MDS [13,14]. Therefore, we recommend establishing the *HFE* gene mutation status (and in the future probably also other iron-storage-related genes) in patients with MDS, at least when these patients present with signs of increased iron uptake before transfusion therapy (elevated serum ferritin, transferrin saturation > 70%), or have a case history of familial haemochromatosis, or have a rapid increase in serum ferritin levels after starting transfusion therapy.

## Prevention of iron overload

A number of effective treatment options are available to treat anaemia and thus help to prevent iron overload and other transfusion-related side-effects in patients with MDS. A direct approach is to administer haematopoietic growth factors, i.e. erythropoietin with or without G-CSF [35–38]. Other drugs act as immunosuppressive agents (lenalidomide, cyclosporine-A, antithymocyte globulin, ATG) or stem cell-targeting therapy (chemotherapy, stem cell transplantation), and thereby may, indirectly, improve or even correct anaemia [3,39–41] (Table 1). An important aspect is that all these drugs act only in a subgroup of patients, which is in line with the notion that MDS represents an extremely heterogeneous group of stem cell neoplasms. Another interesting aspect is that the response to individual drugs can be predicted to a degree in these patients using established predictive parameters. Interestingly, in several instances (e.g. effects of erythropoietin), transfusion dependence itself has been recognized as an important predictive variable concerning the probability of a (erythroid) response [35–38].

**Table 1:** Prevention and therapy of iron overload in MDS: proposed algorithm

Primary considerations:	MDS subtype, IPSS, life expectancy, curative vs. palliative therapy plan, age, comorbidity, mental status
Laboratory investigations:	Haematologic parameters and LDH, serum ferritin, transferrin-saturation, liver enzymes, inflammation parameters, (liver imaging studies, tissue biopsy, preferably bone marrow biopsy)
Prophylactic therapy:	EPO ± G-CSF (Nordic score)
	Lenalidomide (in 5q- syndrome patients)
	ATG + CSA (hypoplastic MDS, HLADR15)
	Demethylating agents (complex karyotype)
	Chemotherapy ± SCT (AML-risk, donor, age)
Established iron overload:	Consider therapy with chelating agents based on the following parameters:
	Serum ferritin > 2000 ng mL^−1^ (without signs of active inflammation or liver disease)
	Transfusion dependent anaemia
	Life expectancy of more than 2 years
	Organopathy resulting from iron overload*
	Planned chemotherapy or SCT†
Selection of chelating agents:	1. Desferoxamine (Desferal®)‡
	2. Deferasirox, ICL670 (Exjade®)‡
	3. Deferiprone, L1 (Ferriprox®)‡

* In these cases, chelating agents should be considered even if the life expectancy is less than two years.

† MDS patients with iron overload who undergo stem cell transplantation have a less favourable outcome (survival) compared to patients without iron overload.

‡ If patients cannot tolerate, or do not respond to Desferal, Exjade should be applied unless kidney function is abnormal. If patients cannot tolerate Exjade, have significant side effects (kidney function) or have no response, Ferriprox or other experimental drugs should be considered (if possible in clinical trials). Abbreviations: MDS, myelodysplastic syndromes; IPSS, international prognostic scoring system; LDH, lactate dehydrogenase; EPO, erythropoietin; G-CSF, granulocyte-macrophage colony-stimulating factor; ATG, antithymocyte globulin; CSA, cyclosporine-A; SCT, stem cell transplantation; AML, acute myeloid leukaemia.

Whatever treatment is considered, early intervention may be the optimal way to prevent iron overload. Erythropoietin (with or without G-CFS) is recommended for low risk MDS patients with transfusion-dependent anaemia in whom endogenous erythropoietin levels and the transfusion-frequency are low [35–38]. Thus, cytokine therapy is usually started in a relatively ‘early’ phase of disease. However, a very early intervention, i.e. before transfusion therapy is initiated, may be questionable for several reasons. First, some of these patients may have a remarkably stable course and stable haemoglobin, at levels that do not require transfusions, and therefore would potentially be ‘overtreated’ when starting too early with growth factors or other, maybe even mutagenic, drugs. Second, most drugs are only approved for transfusion-dependent anaemia. Third, these drugs may also have side effects which should be taken into account in individual patients. Therefore, before starting therapy in non-transfused patients, it may be of great importance to estimate (i) the chance of a patient to develop transfusion-dependence in the near future, (ii) the chance of developing rapid iron-overload, and (iii) the probability of long term AML-free survival. In this regard, it may be of great importance to review the dynamics of anaemia in the past, to study all aspects of the disease including the IPSS, and to ask for signs of emerging iron overload or the genetic risk of developing iron overload.

## Indication for chelation therapy and selection of patients

By consensus, the following groups of patients with MDS should be regarded as candidates for iron chelating therapy:

1. Patients with frank iron overload (e.g. stable/increasing serum ferritin > 2000 ng mL^−1^ without signs of active inflammation or liver disease) who are transfusion-dependent (at any frequency) and have a life expectancy of more than two years.
2. Patients who are transfusion-dependent, receive more than two red cell concentrates per month, at any ferritin level, and have a life expectancy of more than two years (exception: patients with frank iron deficiency, e.g. chronic gastrointestinal tract bleeding).
3. In select cases, iron chelating therapy can also be considered when life expectancy (estimate based on natural history of the disease) is less than two years. Examples are: planned curative therapy (stem cell transplantation), massive iron overload with consecutive organopathy considered to further reduce life expectancy, or massive iron overload that is judged to significantly reduce the quality of life (QOL).

Additional parameters that may influence the decision to treat with iron chelating agents in individual patients with MDS are age (geriatric aspects), social and mental features, comorbidity (organopathy) and the genetic status (*HFE*).

In patients with high ferritin levels (> 2000 ng mL^−1^), iron chelating drugs can also be given, together with other specific drugs like cytokines (erythropoietin ± G-CSF), chemotherapy, or immunomodulating agents, with recognition of potentially harmful drug combination effects (e.g. effects of deferasirox and cyclosporine A, both of which may impair kidney function).

## Selection of chelating agents

Several different iron chelating agents are available, including desferoxamine, deferiprone (L1), and deferasirox (ICL670). Whereas desferoxamine (Desferal®) is usually applied subcutaneously, L1 = deferiprone (Ferriprox®) and ICL670 (Exjade®) are administered orally [3,11]. The historical standard for iron chelation in MDS is desferoxamine [10–12]. However, more and more data suggest that ICL670, and probably L1, can be employed as effective alternative drugs, although it should be pointed out that most of this information stems from trials performed in non-MDS patients [42–46]. The major advantage of these drugs over desferoxamine is their oral use, as continuous subcutaneous infusions are inconvenient and may be associated with local side effects. On the other hand, both deferiprone and deferasirox can also produce relevant side effects. Notably, deferiprone has been described as causing reversible neutropenia in about 5–10% of patients, as well as other side-effects, such as gastrointestinal symptoms, arthropathy, transient changes in liver enzymes and zinc deficiency, and therefore is not considered as an optimal alternative drug for MDS patients who may have pre-existing neutropenia and impaired neutrophil function. Deferasirox has been described as causing (in most cases reversible) impairment of renal function with elevation in serum creatinin levels, and in some cases even fatal irreversible renal failure, as well as other side-effects such as cytopenia, exanthema and an increase in liver enzymes. Therefore, both drugs must be administered with special caution and patients have to be monitored carefully. Likewise, in MDS patients treated with deferasirox, repeated examinations, starting with weekly tests, of the kidney function, including creatinine clearance measurements, should be performed. The various ‘pros’ and ‘cons’ for the use of the three chelators, in general and in MDS, have been discussed recently [47,48]. The major problem in MDS is that only a few studies and data are available for Exjade® so far, and no data on L1/deferiprone are available. Nevertheless, deferasirox (Exjade®) has recently been approved by the FDA and EMEA for the treatment of iron overloaded patients who cannot tolerate or do not respond adequately to desferoxamine.

The current recommendation for therapy of iron overload in MDS is to offer desferoxamine first and to examine the response to this drug. In patients who have no adequate response or are unable to tolerate the drug, we recommend the use of deferasirox (Exjade®) (Table 1) provided that the kidney function (creatinine clearance) is normal, no renal disease is known, and the creatinine clearance remains stable during the first weeks of treatment. Mild changes in kidney function (slight increase in serum creatinine or slight decrease in creatinine clearance) may require dose adjustment (dose reduction), that usually does not lead to a complete loss of response to the drug. If dose reduction is not followed by a return of creatinine levels (creatinine clearance) to normal range, deferasirox should be stopped. In those patients who cannot tolerate or do not respond to Exjade®, or have clear signs of impaired kidney function, Ferriprox® or other experimental drugs (such as triapine or immobilized desferrioxamine; or disease-targeting drugs for the prevention of iron overload) should be offered, if possible in clinical trials. In the case of Ferriprox®, the potential for drug-induced neutropenia should be taken into account. In the case of severe neutropenia (agranulocytosis) or neutropenic infections following deferiprone treatment, the drug should be stopped and G-CSF should be administered.

## Monitoring of response and proposed response criteria

For monitoring iron overload and the response to therapy, we recommend performing serial measurements (every one to 2 months) of serum ferritin levels together with liver enzymes and inflammation parameters. In fact, ferritin is a simple objective test and a relatively reliable and reproducible marker of iron overload unless the patient is suffering from an active hepatic disease or severe chronic inflammation. In such cases, serial imaging studies (MRI, SQUID) may be required. A rebiopsy (bone marrow) is usually not required. Depending on the drug applied, additional parameters need to be monitored, such as kidney function parameters or neutrophil counts.

Unfortunately, no generally accepted response criteria for iron depletion produced by chelating agents in MDS have been defined so far. We recommend the use of the following criteria and the resulting scoring system to measure responses to iron chelating agents in patients with MDS (Table 2).

**Table 2:** Iron overload and therapy: proposed response criteria*

a. **Complete Response** (CR): Decrease of serum ferritin to < 2000 ng mL^−1^*and* decrease at least by 500 ng mL^−1^ (e.g. from 2300 ng mL^−1^ to 1600 ng mL^−1^)
b. **Minor Response** (MiR): Decrease of serum ferritin to < 2000 ng mL^−1^, but less than by 500 ng mL^−1^ (e.g. from 2300 ng mL^−1^ to 1900 ng mL^−1^)
c. **Stable Iron Load**(SIL): Ferritin constantly elevated but < 4000 ng mL^−1^
d. **No Response**(NR): Further increase of ferritin (by at least 500 ng mL^−1^) or ferritin constantly > 4000 ng mL^−1^

* Proposal of the MDS Platform of the Austrian Society for Haematology and Oncology.

A complete response (CR) is defined by a decrease in serum ferritin levels to < 2000 ng mL^−1^ and a decrease by at least 500 ng mL^−1^. If the decrease is less than 500 ng mL^−1^, a minor response (MiR) is reached. In other patients, a stable iron load (SIL) may be observed in the follow up. In these patients, serum ferritin levels are constantly elevated, but are less than 4000 ng mL^−1^. If ferritin levels further increase to > 4000 (and by at least 500 ng mL^−1^) the patient should be classified as a non-responder.

These criteria can be applied in patients receiving chelation therapy, but can also be applied in patients who have received drugs that can lead to transfusion-independence or at least to a decrease in transfusion frequency.

## For how long should chelation be performed?

Currently available data suggest that transfusion-dependent and chelator-responding patients should be kept on iron chelating agents as long as a response can be documented, i.e. as long as no further marked (massive) increase in ferritin levels are seen (i.e. ferritin levels constantly < 4000 ng mL^−1^).

In the case of very high serum ferritin levels or severe organopathy considered to result from iron overload, therapy can also be continued even when the patient is no longer transfusion-dependent (e.g. responds to lenalidomide). These patients should then be treated as long as they are classified as a responder, i.e. serum ferritin is < 4000 ng mL^−1^, or decreases until ferritin has reached a level below 1000 ng mL^−1^. Then, therapy with iron chelating agents should be stopped. If transfusion-dependence develops again in these patients, iron chelating therapy should be reintroduced.

## Concluding remarks and future perspectives

Transfusion-induced iron overload is an emerging clinical problem in patients with MDS. Whereas a subgroup of patients develop iron overload within a short time, others may not require chelation therapy over years despite repeated transfusions. So far, little is known about factors predisposing to iron overload in MDS, about the optimal management of these patients, and about the optimal treatment. The current article provides definitions for iron overload in MDS as well as recommendations for diagnostic investigations and the selection of patients for therapy. In addition, our article proposes therapeutic algorithms as well as response criteria. These recommendations and criteria should help in the management of patients with MDS in practice and in the conduct of clinical trials. In fact, since current recommendations and criteria, including that presented in this article, are mostly based on experience and data from non-MDS trials, prospective trials are warranted to establish the effect of chelation therapy on iron overload and survival in patients with MDS. Until the outcomes of such trials are reported, our proposal and similar recommendations may serve as a useful proposal for the treating physician.

## Address

Department of Internal Medicine I, Division of Haematology & Haemostaseology, Medical University of Vienna, Vienna, Austria (P. Valent, F. Wimazal, W. R. Sperr); First Department of Internal Medicine, Elisabethinen Hospital, Linz, Austria (O. Krieger); Division of Haematology and Oncology, Innsbruck Medical University, Innsbruck, Austria (R. Stauder); Ludwig-Boltzmann Institute for Leukaemia Research and Haematology and 3rd Medical Department, Hanusch Hospital, Vienna, Austria (T. Nösslinger); Division of Haematology, Medical University of Graz, Austria (H. Sill); Institute for Laboratory Medicine, Otto Wagner Hospital Vienna, Austria (P. Bettelheim)
